# Prenatal Exposure to Methamphetamine Causes Vascular Dysfunction in Adult Male Rat Offspring

**DOI:** 10.3389/fcvm.2022.830983

**Published:** 2022-01-26

**Authors:** Hasitha Chavva, Adam M. Belcher, Daniel A. Brazeau, Boyd R. Rorabaugh

**Affiliations:** ^1^Department of Pharmaceutical Sciences, Marshall University School of Pharmacy, Huntington, WV, United States; ^2^Department of Biomedical Sciences, Marshall University School of Medicine, Huntington, WV, United States

**Keywords:** prenatal methamphetamine, perivascular adipose, aorta, vascular, offspring

## Abstract

Methamphetamine use during pregnancy can have negative consequences on the offspring. However, most studies investigating the impact of prenatal exposure to methamphetamine have focused on behavioral and neurological outcomes. Relatively little is known regarding the impact of prenatal methamphetamine on the adult cardiovascular system. This study investigated the impact of chronic fetal exposure to methamphetamine on vascular function in adult offspring. Pregnant female rats received daily saline or methamphetamine (5 mg/kg) injections starting on gestational day 1 and continuing until the pups were born. Vascular function was assessed in 5 month old offspring. Prenatal methamphetamine significantly decreased both the efficacy and potency of acetylcholine-induced relaxation in isolated male (but not female) aortas when perivascular adipose tissue (PVAT) remained intact. However, prenatal methamphetamine had no impact on acetylcholine-induced relaxation when PVAT was removed. Nitroprusside-induced relaxation of the aorta was unaffected by prenatal methamphetamine. Angiotensin II-induced contractile responses were significantly potentiated in male (but not female) aortas regardless of the presence of PVAT. This effect was reversed by L-nitro arginine methyl ester (L-NAME). Serotonin- and phenylephrine-induced contraction were unaffected by prenatal methamphetamine. Prenatal methamphetamine had no impact on acetylcholine-induced relaxation of third order mesenteric arteries and no effect on basal blood pressure. These data provide evidence that prenatal exposure to methamphetamine sex-dependently alters vasomotor function in the vasculature and may increase the risk of developing vascular disorders later in adult life.

## Introduction

According to the 2020 National Survey on Drug Use and Health, approximately 6 % of United States residents over the age of 12 have used methamphetamine at least once during their lifetime (1). Methamphetamine abuse is most prominent among adults 26–49 years of age (1). Notably, this age group includes women of childbearing age. The Infant Development, Environment, and Lifestyle (IDEAL) study estimated that 5.2 % of pregnant women used methamphetamine during their pregnancy (2). Approximately one-third these individuals decreased their methamphetamine use during pregnancy (3). However, most either increased (10 %) or did not change (55 %) their patterns of methamphetamine use between the first and third trimesters of pregnancy, resulting in prenatal exposure of their children to methamphetamine (3).

Prenatal exposure to methamphetamine can lead to adverse neurological consequences in the offspring including an increased prevalence of behavioral and cognitive problems (4, 5), structural changes in the brain (6, 7), and decreased spatial and verbal memory (6). In contrast to the behavioral and neurological consequences, few studies have investigated the impact of prenatal methamphetamine on the cardiovascular function of adult offspring. We previously reported that adult female rats that were prenatally exposed to methamphetamine developed myocardial hypersensitivity to ischemic injury (8). This was accompanied by decreased myocardial expression of protein kinase C-ε and decreased phosphorylation of Akt, proteins that have well established roles in protecting the heart from ischemia. Importantly, these changes did not occur in the hearts of their adult male littermates, demonstrating that prenatal exposure to methamphetamine can induce sex-dependent changes in the heart that persist into adulthood.

Other investigators have reported that prenatal exposure to cocaine induces sex-dependent changes in the vasculature of adult offspring including potentiation of norepinephrine-induced vasoconstriction, increased myofilament sensitivity to calcium, attenuation of endothelium-dependent relaxation, and suppression of the baroceptor reflex (9). Prenatal cocaine also impairs myogenic reactivity of the coronary arteries in adult offspring (10). Cocaine and methamphetamine have different mechanisms of action. However, both stimulants act on central and peripheral neurons to increase adrenergic and dopaminergic signaling, leading to similar physiological effects. In contrast to prenatal cocaine, it is unknown whether the adult vasculature is impacted by prenatal exposure to methamphetamine. The goal of the present study was to determine whether prenatal methamphetamine alters vascular function in adult offspring. In light of recent studies demonstrating that perivascular adipose tissue (PVAT) plays an important role in the regulation of vascular tone (11–14), the impact of prenatal methamphetamine was investigated both in the presence and absence of PVAT.

## Methods

### Animals

Male and female Sprague-Dawley rats purchased from Charles River labs (strain code 001) were co-housed in standard cages with free access to food and water and a 12/12 hr light/dark cycle (lights on at 0600). Gestational day 0 was defined as the day of detection of a vaginal plug. Pregnant dams were administered saline or methamphetamine (5 mg/kg/day) by subcutaneous injection once per day (at 0800) starting on gestational day 1 and continuing until the pups were born. Pups were weaned at 28 days of age and housed two animals per cage. Blood pressure and vasomotor responses in isolated aortas were measured when the animals reached 5 months of age. Vasomotor responses in isolated third order mesenteric arteries were measured in 6 month old animals. All procedures were approved by the Institutional Animal Care and Use Committee.

### Measurement of Vasomotor Responses in Isolated Aorta

Male and female rats were deeply anesthetized with pentobarbital (100 mg/kg), and the thoracic aorta was removed. Aortas were cut into rings 2-mm in length. Two aortic rings were used from each rat. The perivascular adipose tissue (PVAT) of one ring was left intact, and the PVAT was removed from the other ring. The tissues were mounted on a Radnoti 4-channel tissue bath system (Radnoti LLC, Monrovia, CA) in chambers filled with 10 ml Krebs solution (in mM: 118 NaCl, 4.7 KCl, 1.2 MgSO_4_, 25 NaHCO_3_, 1.2 KH_2_PO_4_, 0.5 Na_2_EDTA, 11 glucose, and 2.5 CaCl_2_, pH 7.4) bubbled with 95% O_2_ and 5% CO_2_ at 37°C. The tissue was equilibrated for one h under a resting tension of 3 g. Krebs solution was changed every 15 min during the equilibration phase.

Tissue viability was verified by measuring the contractile response to 60 mM potassium chloride (KCl). Two KCl responses (each 10 min in duration) were recorded and the second KCl response was used to normalize the subsequent contractile responses to phenylephrine, serotonin, and angiotensin II. Tissues that did not contract in response to 60 mM KCl were not used for further study. Cumulative concentration response curves were generated in two different sets of rats. In the first set of animals, phenylephrine induced contractile responses were measured followed by acetylcholine-induced relaxation of the phenylephrine-contracted vessels. This was followed by serotonin-induced contractile responses in the same tissue. A second set of animals was used to measure angiotensin-II-induced contraction and nitroprusside-induced relaxation. Two responses to 60 mM KCl (each 10 min in duration) were recorded. After thoroughly washing the KCl from the tissue, responses to cumulative concentrations of angiotensin II were measured. After angiotensin II was washed from the tissue, the aortic rings were precontracted with 10 μM phenylephrine prior to measuring sodium nitroprusside-induced relaxation. Contractile responses induced by phenylephrine and angiotensin-II were normalized to the response generated by 60 mM KCl. Acetylcholine and nitroprusside-induced relaxation responses were normalized to the tension induced by precontraction with 10 μM phenylephrine. All data were recorded with a Power Lab 4/30 data acquisition system (AD Instruments, Colorado Springs, CO) using LabChart 8 (AD instruments, Colorado Springs, CO) software.

### Vasomotor Responses in Third Order Mesenteric Arteries

Third order mesenteric arteries (~250 μm in diameter) were isolated from 6 month old male rats and placed in calcium-free physiological saline solution (PSS) (in mM: 130 NaCl, 4.7 KCl, 1.18 KH_2_PO_4_, 1.17 MgSO_4_, 24.9 NaHCO_3_, 5.5 glucose, 0.026 EDTA; pH 7.4). Mesenteric rings (with PVAT either attached or removed) were cut to a length of 1.8 mm and mounted in a prewarmed (37° C) 4-channel wire myograph (DMT 620M, Danish Myo Technology, Denmark), chamber filled with PSS containing 40 mM CaCl_2_ that was continuously aerated with 95 % O_2_/5 % CO_2_. Contractile function was continuously recorded by a Power Lab 4/30 data acquisition system (AD Instruments, Colorado, US) using Lab Chart Pro v8.1.19 (AD Instruments, Colorado, US) software. Mounted vessels were normalized by using the micrometer to progressively stretch the tissue until it reached a wall tension equivalent to 100 mmHg of pressure (11, 12). The vessels were then prepared for experimentation using a “wake-up” protocol in which the tissue was contracted with physiological saline containing high potassium (KPSS) (in mM: 74.7 NaCl, 60 KCl, 1.18 KH_2_PO_4_, 1.17 MgSO_4_, 24.0 NaHCO_3_, 5.5 glucose, 0.026 EDTA, 1.6 CaCl_2_) and then washed three times. The tissue was then contracted with 10 μM norepinephrine and washed three times. Functionality of the tissue was tested by precontracting the vessels with 1 μM norepinephrine followed by relaxation of the precontracted tissue with 10 μM acetylcholine. Vessels that failed to contract to 1 μM norepinephrine or to relax in response to 10 μM acetylcholine were not used for further experiments. The vessels were then contracted with KPSS for 10 min. The tissue was washed three times and then contracted with 10 μM phenylephrine prior to measuring relaxation with cumulative concentrations of acetylcholine. The tissue was then washed three more times before precontracting the tissue with 10 μM phenylephrine and measuring relaxation in response to cumulative concentrations (10 pM−10 μM) of sodium nitroprusside. Relaxation responses to acetylcholine and nitroprusside were normalized to precontraction tension induced by 10 μM phenylephrine.

### Measurement of Blood Pressure

Blood pressure was measured by tail cuff plethysmography using the CODA® System (Kent Scientific Corporation, Torrington, CT) according to the manufacturer's instructions. Blood pressure was measured in each animal for five consecutive days. The first three days enabled the animals to become acclimated to the procedure. Duplicate measurements on days 4 and 5 were averaged to represent the blood pressure of each individual animal. All blood pressure measurements were performed in the morning between 0900 and 1200.

### Western Blots

PVAT was isolated from aortas of 5 month old male offspring that had been prenatally exposed to saline or methamphetamine (5 mg/kg) as described above. The tissue was homogenized in 500 μL solubilization buffer [150 mM sodium chloride, 50 mM Tris (pH 8.0), 1% NP-40, 0.5% sodium deoxycholate, 0.1% sodium dodecyl sulfate, 10 μM E-64 protease inhibitor, and 1 % Sigma phosphatase inhibitor cocktail 3 (Sigma Cat # P0044)] using a polytron and then centrifuged for 15 min at 14, 000 × G at 4 °C. The supernatant was transferred to a separate tube (being careful to avoid the pellet at the bottom and the lipids at the top) and recentrifuged. The supernatant was transferred to a third tube. After measuring the protein concentration of each sample, the homogenates were diluted in loading buffer (such that the protein concentration in each sample was 2 μg/μL) and boiled for 5 min. The protein (30 μg) was separated on a 10% polyacrylamide electrophoresis gel and then transferred to a nitrocellulose membrane. The membrane was blocked with 5% nonfat dry milk and blotted with antibodies for eNOS and glyceraldehyde-3-phosphate dehydrogenase (GAPDH) (Cell Signaling Technology, Danvers, MA; antibody # 32027 and # 2118, respectively) overnight. The blots were then incubated with a horseradish peroxidase-linked anti-rabbit antibody (Cell Signaling Technology antibody # 7074). Bands were quantified using ImageJ software, and band intensities for eNOS were normalized to those of GAPDH.

### Statistical Analysis

Concentration response curves were analyzed by non-linear regression. pEC_50_ values and the magnitude of agonist-induced contractile or relaxation responses were analyzed by two-way ANOVA with treatment (prenatal saline vs. prenatal methamphetamine) and the presence or absence of PVAT as factors and Tukey's *post-hoc* analysis. Concentration response curves for male and female vessels were analyzed separately. Blood pressure was analyzed by two-way ANOVA (factors = sex and prenatal treatment) and Tukey's *post-hoc* analysis. *P* values ≤ 0.05 were considered statistically significant. Graphpad prism 8 (San Diego, CA) software was used for all the analyses. All results are expressed as mean ± SEM.

## Results

### Maternal Weight Gain, Litter Sizes, and Body Weights of 28 Day Old Offspring

**Figure 1:**
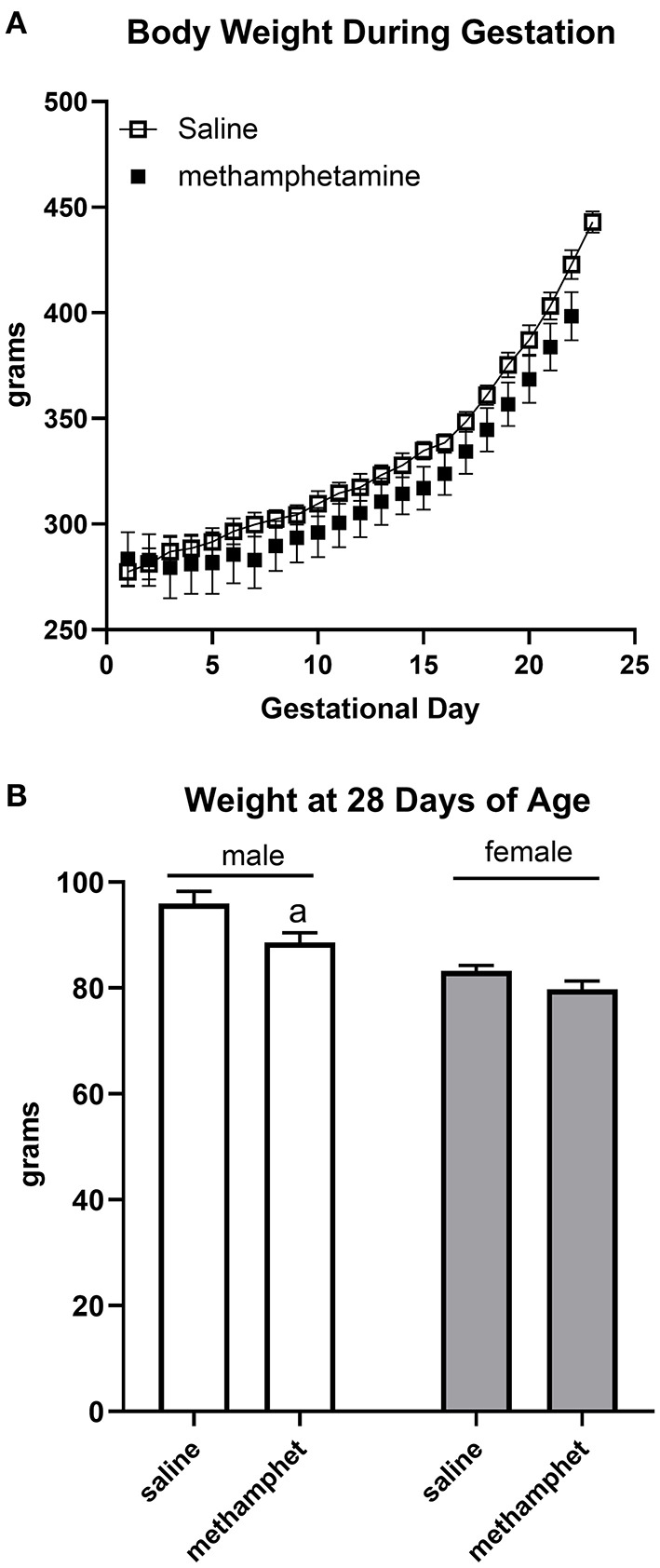
Impact of methamphetamine on gestational weight and body weight of offspring. Pregnant female rats received daily injections of saline (*n* = 5) or methamphetamine (5 mg/kg) (*n* = 5) starting on gestational day 1 and continuing until the pups were born. Two-way ANOVA indicated a significant effect of time [F = 140 (2, 11), *p* < 0.0001] but no effect of methamphetamine on gestational body weight. All methamphetamine-treated dams delivered pups on gestational day 22. Three saline treated dams delivered pups on gestational day 22 and two dams delivered on day 23. Data represent the mean ± SEM of 5 pregnant dams in each group **(A)**. Offspring were weighed at 28 days of age at the time of weaning. Two way ANOVA indicated significant effects of sex [F = 37 (1, 97), *p* < 0.0001] and methamphetamine treatment [F = 9 (1, 97), *p* < 0.005] on body weight in 28 day old offspring. Data represent the mean ± SEM of 21–30 offspring in each group. ^a^indicates *p* < 0.05 compared to male offspring treated with prenatal saline **(B)**.

Female rats that were treated with saline or methamphetamine had similar body weights on gestational day 1 (288 ± 3 and 290 ± 14 for saline or methamphetamine treated dams, respectively). Two-way ANOVA indicated a significant effect [F = 140 (2, 11), *p* < 0.0001] of time (gestational day) on body weight, but there was no significant effect of methamphetamine (Figure 1A). All dams delivered pups on gestational day 22 except for two saline-treated dams that delivered on day 23. Litter sizes were similar in saline (13 ± 0.3) and methamphetamine-treated dams (12 ± 1). There was a significant effect [F = 9 (1, 97), *p* < 0.005] of methamphetamine on body weight when pups were weaned at 28 days of age. *Post-hoc* analysis indicated that body weights of male pups (but not female pups) were significantly smaller following treatment with prenatal methamphetamine compared to those treated with prenatal saline (Figure 1B). However, there was no effect of methamphetamine on body weight in either male (578 ± 10 and 583 ± 10 g for saline and methamphetamine, respectively) or female (308 ± 6 and 326 ± 25 g for saline and methamphetamine, respectively) offspring when the animals were sacrificed at 5 months of age.

### Sex-Dependent Impairment of Acetylcholine-Induced Relaxation of the Aorta Following Prenatal Exposure to Methamphetamine

**Figure 2:**
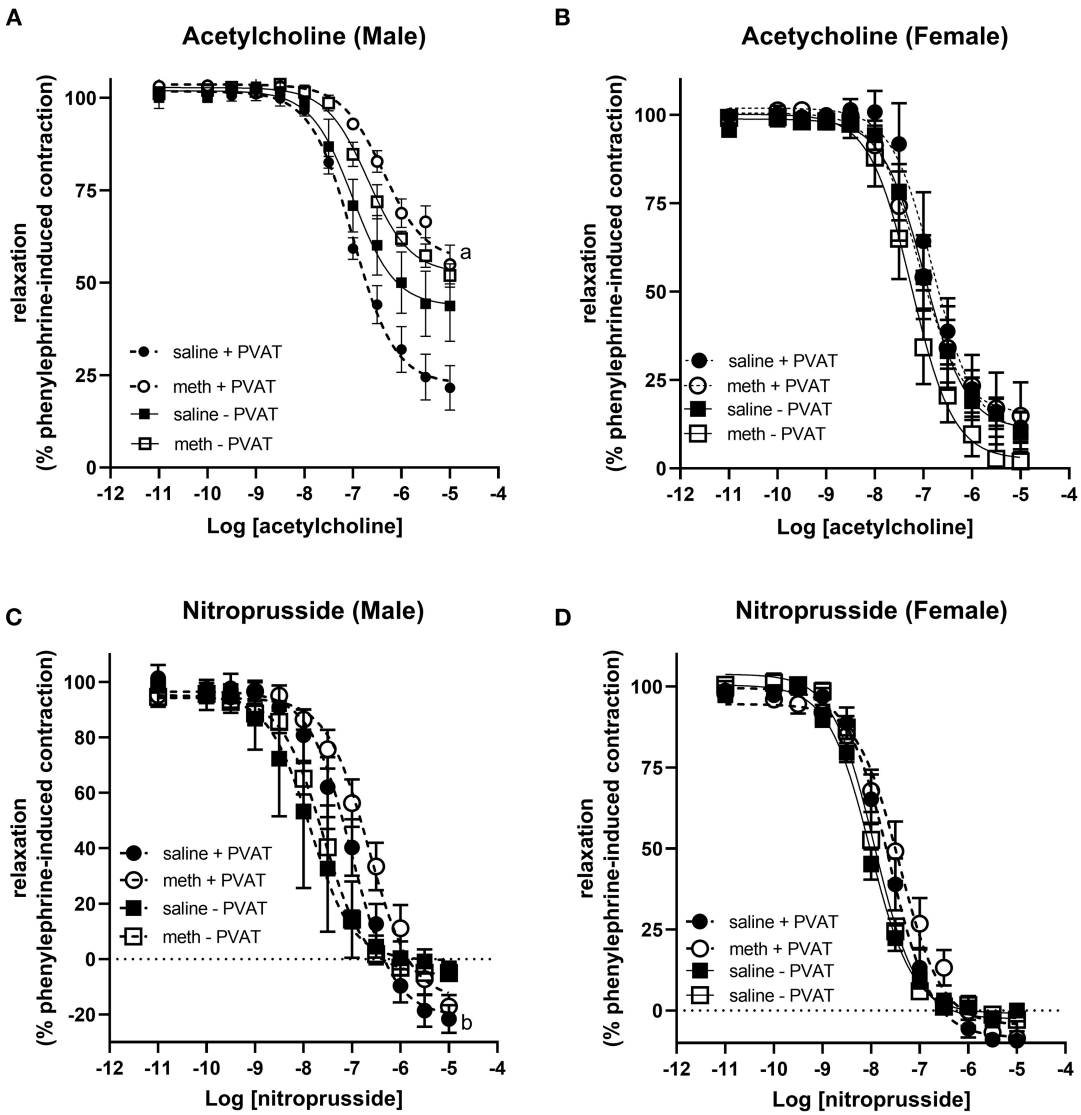
Prenatal methamphetamine causes sex-dependent dysfunction of perivascular adipose tissue in adult aortas. Acetylcholine- and nitroprusside-induced relaxation was measured in the aortas of adult (5 months old) offspring in the presence or absence of PVAT. Two-way ANOVA indicated a significant effect of prenatal methamphetamine [F = 11 (1,21), *p* < 0.005] and a significant interaction between PVAT and methamphetamine [F = 5 (1, 21), *p* < 0.05] on the pIC50 of acetylcholine-induced relaxation in male offspring **(A)**. There was also a significant effect of methamphetamine on the maximal response [F = 8 (1,21), p = 0.01] induced by acetylcholine in male offspring **(A)**. However, prenatal treatment with methamphetamine had no impact on acetylcholine-induced relaxation in female aortas **(B)**. Prenatal methamphetamine had no effect on nitroprusside-induced relaxation in male **(C)** or female **(D)** aortas. ^a^indicates a significant difference (*p* < 0.05) compared to saline + PVAT. ^b^ indicates a significant difference (*p* < 0.05) compared to saline – PVAT.

Acetylcholine induced vasodilation was measured in aortic rings from which the perivascular adipose tissue (PVAT) had been removed and in rings in which the PVAT was intact. Prenatal exposure to methamphetamine significantly decreased both the efficacy and potency of acetylcholine-induced relaxation in male aortas when PVAT was intact (Figure 2A, Table 1). However, prenatal methamphetamine had no impact on acetylcholine-induced relaxation when PVAT was removed from male aortas (Figure 2A, Table 1). In contrast to male aortas, prenatal methamphetamine had no impact on the acetylcholine-induced relaxation in female aortas regardless of the presence of PVAT (Figure 2B, Table 2).

**Table 1:** Acetylcholine- and nitroprusside-induced relaxation responses in aortas isolated from adult male rats that were exposed to fetal saline or methamphetamine (5 mg/kg/day) throughout the gestational period.

		**pIC50**		**Max Relaxation**	
		**Saline**	**Methamphetamine**	**Saline**	**Methamphetamine**
Acetylcholine	+PVAT (*n =* 5–7)	–7.0 ± 0.1	–6.3 ± 0.1^a^	22 ± 6	55 ± 5^a^
	– PVAT (*n =* 5–8)	–6.8 ± 0.2	–6.9 ± 0.1	39 ± 10	53 ± 3
Nitroprusside	+PVAT (*n =* 6)	–7.1 ± 0.2	–6.7 ± 0.2	–21 ± 5	–17 ± 6
	– PVAT (*n =* 6)	–7.9 ± 0.2^a^	–7.6 ± 0.1^b^	–3 ± 2^a^	–6 ± 3

*Two-way ANOVA indicated a significant effect of prenatal methamphetamine [F = 11 (1,21), p <0.005] and a significant interaction between PVAT and methamphetamine [F = 5 (1, 21), p <0.05] on the pIC50 of acetylcholine-induced relaxation. There was also a significant effect of methamphetamine on the maximal response [F = 8 (1,21), p = 0.01] induced by acetylcholine. Two-way ANOVA indicated a significant effect of PVAT on the pIC50 [F = 24(1,22), p <0.0001] and maximal response [F = 15(1,22), p <0.005] of nitroprusside-induced relaxation*.

a *indicates a significant difference (p <0.05) compared to saline + PVAT*.

b *indicates a significant difference (p <0.005) compared to methamphetamine + PVAT*.

**Table 2:** Acetylcholine- and nitroprusside-induced relaxation responses in aortas isolated from adult female rats that were exposed to fetal saline or methamphetamine (5 mg/kg/day) throughout the gestational period.

		**pIC50**		**Max Relaxation**	
		**Saline**	**Methamphetamine**	**Saline**	**Methamphetamine**
Acetylcholine	+ PVAT (*n =* 6)	–6.8 ± 0.2	–7.1 ± 0.2	8 ± 4	13 ± 10
	– PVAT (*n =* 6)	–6.9 ± 0.2	–7.3 ± 0.2	9 ± 5	1 ± 6
Nitroprusside	+ PVAT (n= 6)	–7.7 ± 0.1	–7.5 ± 0.2	–8 ± 2	–4 ± 4
	– PVAT (*n =* 6)	–7.9 ± 0.1	–8.1 ± 0.01^a^	–3 ± 2	–1± 2

*Two-way ANOVA indicated a significant effect of PVAT on pIC50 [F = 18 (1,19), p <0.0005] of nitroprusside-induced relaxation*.

a *Indicates p <0.05 compared to methamphetamine + PVAT*.

Nitroprusside-induced relaxation was measured to determine whether methamphetamine-induced impairment of acetylcholine-induced relaxation in male aortas was a consequence of impaired nitric oxide (NO) synthesis or decreased sensitivity of vascular smooth muscle to NO. Two way ANOVA indicated a significant effect of PVAT on the pEC50 [F = 24 (1, 22), *p* < 0.0001] and maximal relaxation [F = 15 (1, 22), *p* < 0.001] in male aortas (Figure 2C, Table 1). There was also a significant effect of PVAT on the pEC50 of nitroprusside in female aortas [F = 18 (1, 19), *p* < 0.0005] (Figure 2D, Table 2). In fact, the presence of PVAT caused both male and female aortas to relax below the baseline level of tension that existed prior to phenylephrine-induced precontraction (below 0). However, prenatal exposure to methamphetamine had no impact on either the pEC50 or the maximal relaxation induced by nitroprusside in aortas from either sex.

### Sex-Dependent Potentiation of Angiotensin II-Induced Contractile Responses in the Aorta Following Prenatal Exposure to Methamphetamine

**Figure 3:**
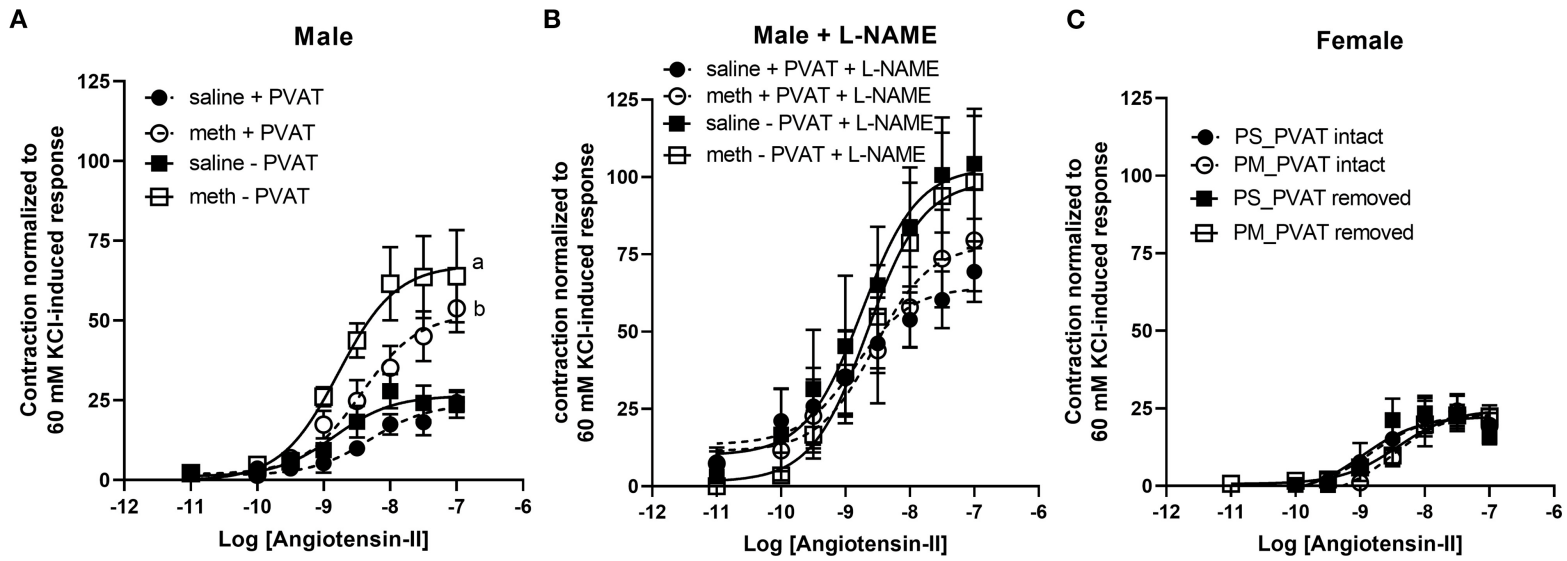
Prenatal methamphetamine sex-dependently potentiates angiotensin II-induced contraction of isolated aortas. The magnitude of angiotensin II-induced contractile responses were significantly [F = 15 (1, 25), *p* < 0.001] potentiated in aortas from adult male rats that had been prenatally treated with methamphetamine compared to aortas from males that had been prenatally treated with saline regardless of whether PVAT was present **(A)**. Prenatal exposure to methamphetamine had no impact on angiotensin II-induced contractile responses in the presence of 100 μM L-NAME. The magnitude of angiotensin II-induced contraction was significantly [F = 4 (1, 20), *p* = 0.05] increased by PVAT in the presence of L-NAME, but *post-hoc* analysis did not indicate differences between specific groups **(B)**. Angiotensin II-induced-responses in female aortas were unaffected by prenatal methamphetamine **(C)**. ^a^indicates a significant difference (*P* < 0.05) compared to aortas from saline treated rats in the absence of PVAT (– PVAT), and ^b^indicates a significant difference (*p* < 0.05) compared to aortas from saline treated rats with the PVAT intact (+ PVAT). Data represent the mean ± SEM of 6–8 separate animals.

Contractile responses to angiotensin II, phenylephrine, and serotonin were measured in aortas from adult male and female rats following prenatal exposure to saline or methamphetamine. The efficacy of angiotensin II was significantly [F = 15 (1, 25), *p* < 0.001] potentiated by prenatal exposure to methamphetamine in adult male rats regardless of the presence or absence of PVAT (Figure 3A, Table 3). This effect was blocked by L-NAME (Figure 3B, Table 3). In contrast to male aortas, prenatal methamphetamine did not alter the efficacy of angiotensin II induced contractile responses in female aortas (Figure 3C, Table 4). Two way-ANOVA indicated a significant [F = 6 (1, 20), *p* < 0.05] overall effect of prenatal methamphetamine on the pEC50 values of angiotensin II-induced contraction in female aortas (Table 2). However, *Post-hoc* analysis did not detect any significant differences between specific groups of female aortas.

**Table 3:** Angiotensin II, phenylephrine, and serotonin-induced contractile responses in aortas isolated from adult male rats that were exposed to fetal saline or methamphetamine (5 mg/kg/day) throughout the gestational period.

		**pEC50**		**Max contraction**	
		**Saline**	**Methamphetamine**	**Saline**	**Methamphetamine**
Angiotensin II	+PVAT (*n =* 7–8)	–8.4 ± 0.2	–8.3 ± 0.2	24 ± 4	59 ± 9.0^a^
	– PVAT (*n =* 6–7)	–8.8 ± 0.1	–9.0 ± 0.2	27 ± 5	62 ± 14^a^
Angiotensin II + L-NAME	+PVAT (*n =* 6)	–8.8 ± 0.3	–8.4 ± 0.2	70 ± 8	81.4 ± 10
	– PVAT (*n =* 6)	–8.7 ± 0.2	–8.6 ± 0.1	95 ± 15	92.6 ± 16
Phenylephrine	+PVAT (*n =* 5–6)	–6.9 ± 0.1	–7.2 ± 0.1	130 ± 7	143 ± 7
	– PVAT (n =5)	–7.2 ± 0.2	–7.4 ± 0.04	135 ± 2	150 ± 10
Serotonin	+PVAT (*n =* 5)	–5.1 ± 0.1	–4.7 ± 0.2	128 ± 11	149 ± 10
	– PVAT (*n =* 6)	–5.3 ± 0.1	–5.0 ± 0.1	189 ± 9^b^	180 ± 11

*Two-way ANOVA indicated a significant effect of methamphetamine [F = 15 (1, 25), p = 0.001] on the maximal response to angiotensin II and a significant effect of PVAT [F = 8 (1, 25) p <0.01] on the pEC50 of angiotensin II-induced contraction. Two-way ANOVA indicated significant effects of methamphetamine on pEC50 [F = 5 (1, 18), p <0.05] and PVAT [F = 21 (1, 18), p <0.0005] on the maximal contractile response to serotonin*.

a *indicates a significant difference (p <0.05) compared to aortas from saline-treated rats with perivascular adipose tissue intact (+ PVAT)*.

b *iindicates a significant difference (p <0.005) compared to aortas from saline-treated rats + PVAT*.

**Table 4:** Angiotensin II, phenylephrine, and serotonin-induced contractile responses in aortas isolated from adult female rats that were exposed to fetal saline or methamphetamine (5 mg/kg/day) throughout the gestational period.

		**pEC50**		**Max contraction**	
		**Saline**	**Methamphetamine**	**Saline**	**Methamphetamine**
Angiotensin II	+ PVAT (*n =* 6)	–8.6 ± 0.2	–8.4 ± 0.1	25 ± 6	25 ± 6
	– PVAT (*n =* 6)	–8.9 ± 0.1	–8.4 ± 0.1	23 ± 3	25 ± 4
Phenylephrine	+ PVAT (*n =* 6)	–6.7 ± 0.2	–6.8 ± 0.1	118 ± 10	118 ± 11
	– PVAT (*n =* 6)	–7.1 ± 0.04^a^	–7.0 ± 0.1	128 ± 6	123 ± 6.5
Serotonin	+ PVAT (*n =* 3–4)	–5.1 ± 0.3	–5.4 ± 0.2	163 ± 18	153 ± 26
	– PVAT (*n =* 4)	–5.3 ± 0.2	–5.4 ± 0.2	147 ± 8	152 ± 5

*Two-way ANOVA indicated a significant effect of prenatal methamphetamine [F = 6 (1, 20), p <0.05] on the pEC50 of angiotensin II-induced contraction*.

a *p <0.05 compared to saline +PVAT. There was also a significant effect of PVAT on the pEC50 [F = 11 (1, 20), p <0.005] of phenylephrine-induced contraction. However, Post-hoc analyses indicated that there were no significant effects between specific groups*.

### Prenatal Methamphetamine Has no Impact on Phenylephrine or Serotonin-Induced Contraction

**Figure 4:**
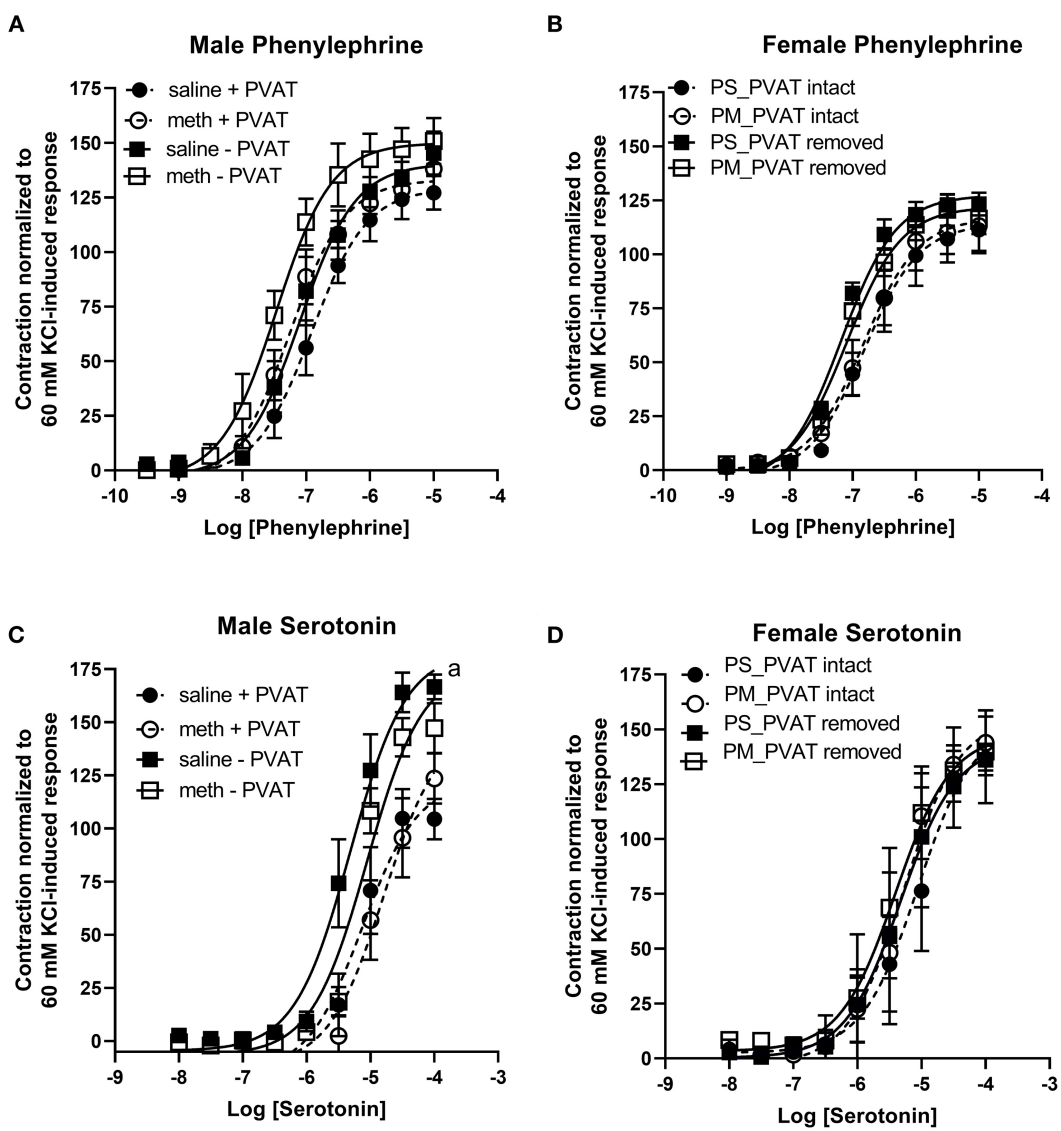
Phenylephrine- and serotonin-induced contractile responses in male and female aortas following prenatal treatment with saline or methamphetamine. Neither fetal exposure to methamphetamine nor the presence of PVAT influenced phenylephrine-induced contraction in male **(A)** or female **(B)** aortas. Two way ANOVA indicated a significant effect [F = 5 (1, 18), *p* < 0.05] of methamphetamine on the pEC50 of serotonin-induced contraction in male aortas **(C)** and a significant effect [F = 21 (1, 18), *p* < 0.0005] of PVAT on the maximal contraction response to serotonin in male aortas **(C)**. Prenatal exposure to methamphetamine and the presence of PVAT had no impact on serotonin-induced contractile responses in female aortas **(D)**. ^a^indicates a significant difference (*p* < 0.005) in the maximal response compared to aortas from saline treated rats with PVAT removed (- PVAT). Data represent the mean ± SEM of 5–6 male animals and 3–6 female animals.

Prenatal methamphetamine had no significant impact on phenylephrine- or serotonin- induced contractile responses in male (Figures 4A,C, Table 3) or female (Figures 4B,D, Table 4) aortas. The efficacy of serotonin was significantly enhanced by the removal of PVAT in male aortas (Figure 4C, Table 3), and the pEC50 of phenylephrine was significantly increased by the removal of PVAT in female aortas (Figure 4, Table 4). However, prenatal exposure to methamphetamine had no significant impact on either the efficacy or potency (pEC50) of phenylephrine or serotonin- induced contraction.

### Prenatal Methamphetamine Has no Effect on Endothelial Nitric Oxide Synthase (ENOS) Expression in PVAT

**Figure 5:**
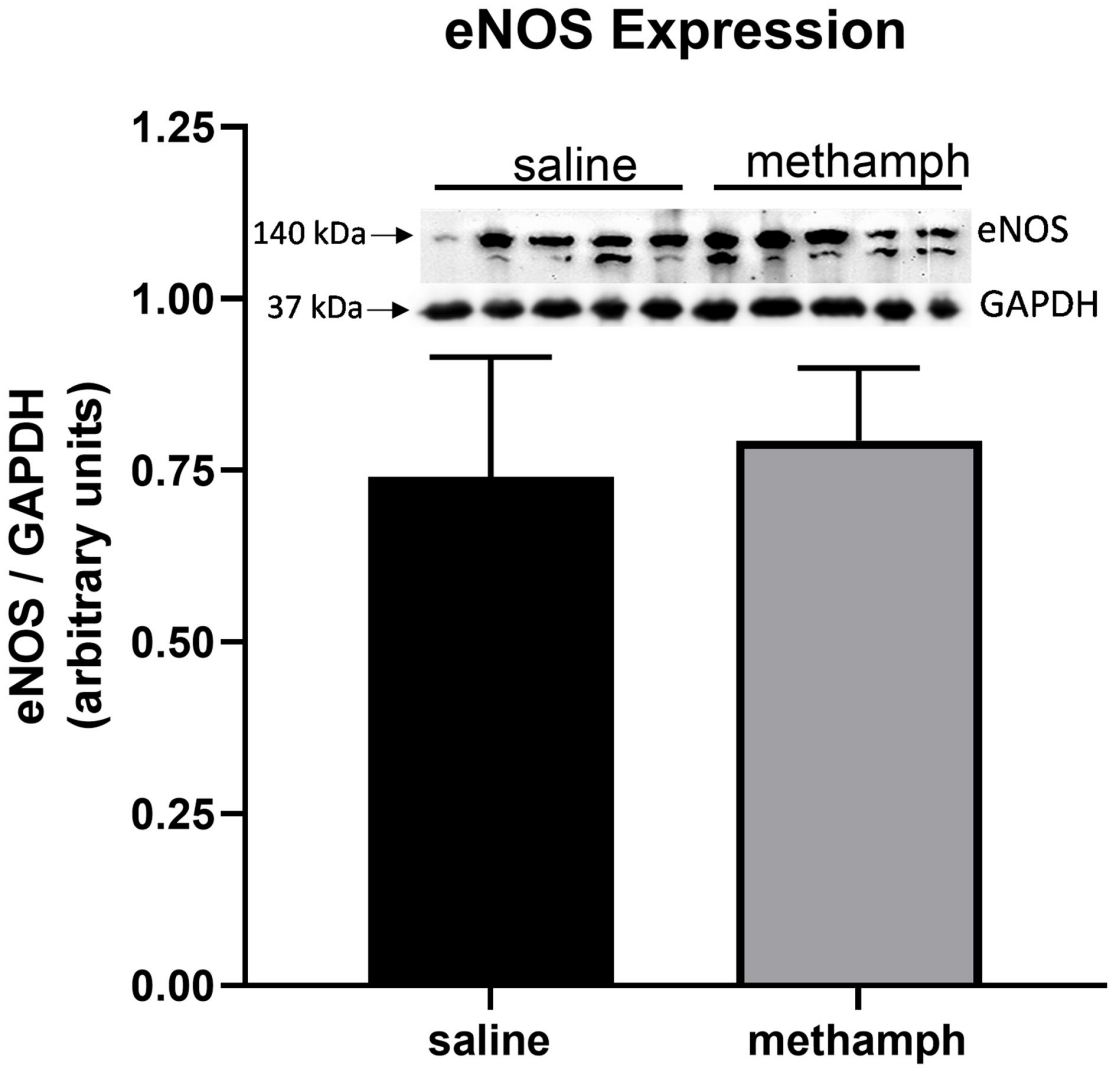
Prenatal methamphetamine exposure had no effect on eNOS expression in male PVAT. Female rats received daily subcutaneous injections of methamphetamine (5 mg/kg) or saline starting on gestational day 1 and continuing until the pups were born. PVAT was isolated from the aortas of male offsping and analyzed by western blotting for endothelial nitric oxide synthase (eNOS) and glyceraldehyde-3-phosphate dehydrogenase (GAPDH). Band intensities were quantified by NIH Image J software. eNOS expression was normalized to that of GAPDH. Bars represent the mean ± S.E.M of PVAT from 5 different male rats.

Western blotting was used to measure eNOS expression in PVAT isolated from male aortas. eNOS expression was unaffected by prenatal exposure to methamphetamine (Figure 5). We made multiple attempts to measure phosphorylation of eNOS at Ser^1177^ and Thr^495^ but were unable to detect phosphorylation at these residues (data not shown).

### Prenatal Methamphetamine Has no Effect on Vascular Responses in Mesenteric Resistance Arteries

**Figure 6:**
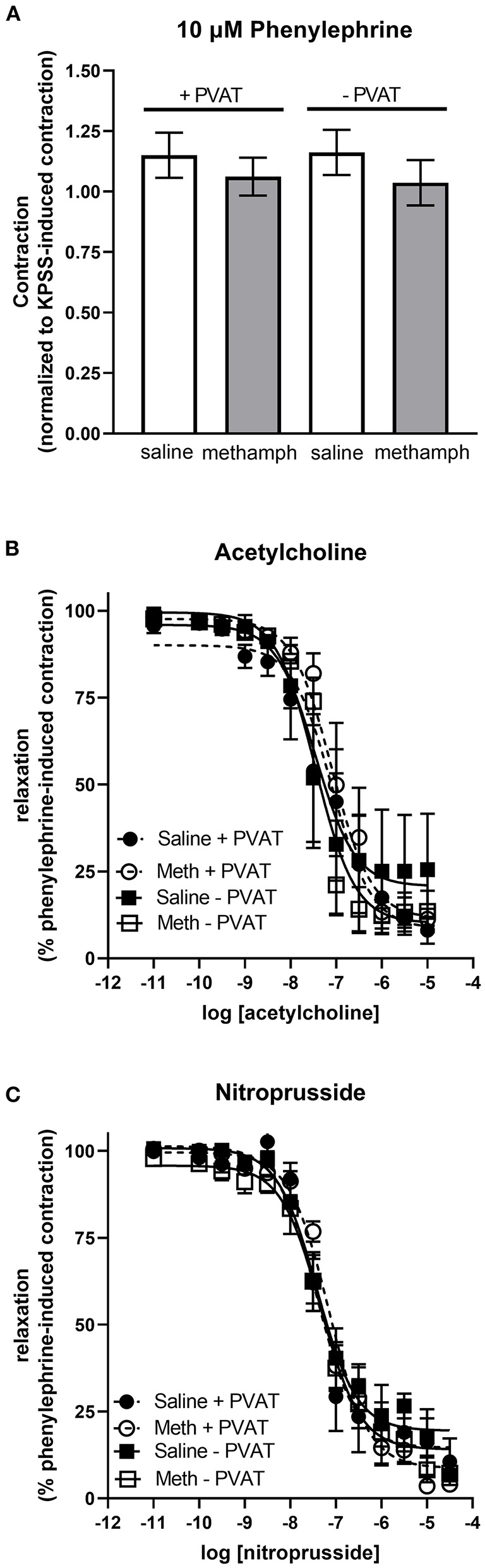
Impact of prenatal methamphetamine on acetylcholine and nitroprusside induced relaxation in third order mesenteric arteries from adult male offspring. Two-way ANOVA indicated no significant effect of prenatal methamphetamine or perivascular adipose tissue (PVAT) on the magnitude of phenylephrine (10 μM)-induced precontraction of mesenteric resistance arteries **(A)**. Acetylcholine **(B)** and nitroprusside **(C)** induced relaxation of these vessels was also unaffected by prenatal exposure to methamphetamine. Data represent the mean ± SEM of 4-6 separate animals.

Precontraction of third order mesenteric arteries with 10 μM phenylephrine produced similar contractile responses regardless of whether the animals had been prenatally treated with saline or methamphetamine and regardless of whether PVAT was present (Figure 6A). Prenatal methamphetamine had no effect on acetylcholine- or nitroprusside-induced induced relaxation of these vessels regardless of the presence or absence of PVAT (Figures 6B,C, Table 5). We attempted to measure angiotensin II-induced contractile responses in these vessels but were unable to achieve consistent responses.

**Table 5:** Acetylcholine- and nitroprusside-induced relaxation responses in third order mesenteric arteries isolated from adult male rats following prenatal exposure to saline or methamphetamine (5 mg/kg/day) throughout the gestational period.

		**pIC50**		**Max relaxation**	
		**Saline**	**Methamphetamine**	**Saline**	**Methamphetamine**
Acetylcholine	+ PVAT (*n =* 4–5)	–7.2 ± 0.4	–7.0 ± 0.2	6 ± 4	7 ± 3
	– PVAT (*n =* 3–5)	–7.7 ± 0.2	7.3 ± 0.1	4 ± 2	10 ± 5
Nitroprusside	+ PVAT (n= 4–5)	–7.4 ± 0.2	–7.1 ± 0.1	4 ± 1	9 ± 4
	– PVAT (*n =* 4–5)	–7.3 ± 0.1	–7.3 ± 0.1	17 ± 3	14 ± 4

### Effect of Prenatal Methamphetamine on Basal Blood Pressure

**Figure 7:**
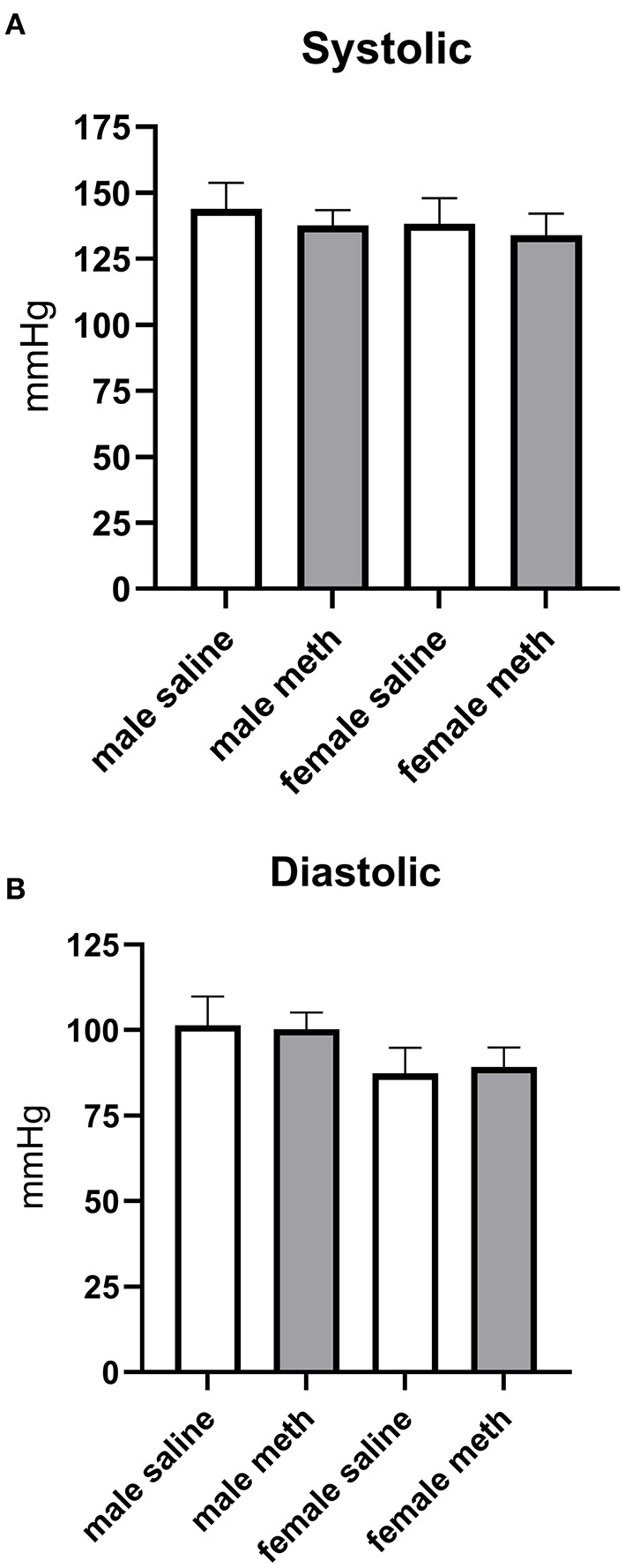
Prenatal exposure to methamphetamine had no impact on basal systolic **(A)** or diastolic **(B)** blood pressure in male or female offspring. Data represent the mean ± SEM of 6–8 separate animals.

Blood pressure was measured by tail cuff plethysmography. Prenatal exposure to methamphetamine had no significant effect on basal systolic or diastolic blood pressures in either male or female rats (Figure 7).

## Discussion

The behavioral and neurological deficits induced by prenatal exposure to methamphetamine have been well established (4–6, 13). In contrast, the impact of prenatal methamphetamine exposure on cardiovascular function in adult offspring has been virtually ignored. We previously reported that prenatal exposure to methamphetamine sex dependently sensitizes the adult heart to ischemic injury (8). The present investigation provides evidence that methamphetamine use during pregnancy also alters the vascular function of adult offspring in a sex-dependent manner.

Historically, most studies investigating the contractility of isolated vessels have been performed in the absence of PVAT. PVAT was usually dissected from isolated vessels prior to measuring contractile responses because it was thought to provide structural support for the vasculature without contributing to vascular contractile function. More recently, PVAT has become well recognized as an important modulator of vascular tone (14–17). PVAT surrounds most blood vessels and serves as a source of NO, adipokines and other paracrine factors that promote arterial relaxation (14–17). It is now understood that signaling molecules released from PVAT regulate constriction and dilation of the vasculature and that PVAT dysfunction may play a role in vascular diseases such as obesity-induced hypertension, atherosclerosis, and cardiometabolic disorders (15, 17, 18).

Our data indicate that prenatal methamphetamine alters vascular function through both PVAT-dependent and PVAT-independent mechanisms. Attenuation of acetylcholine-induced relaxation was PVAT-dependent. In contrast, potentiation of angiotensin II-induced contraction occurred independent of PVAT. Other investigators have reported that cholinergic receptors located in PVAT are coupled to eNOS and that NO originating from PVAT contributes to relaxation of blood vessels following cholinergic receptor stimulation (14). Consistent with this prior work, we found that acetylcholine-induced relaxation of the aorta was completely abolished by L-NAME regardless of whether or not PVAT was present (data not shown). eNOS phosphorylation at Ser^1177^ and Thr^495^ has been used by others as an indicator of eNOS activity. However, recent work demonstrating that eNOS phosphorylation at these residues does not correlate with NO formation has called into question the use of eNOS phosphorylation as a surrogate marker for NO production (19). Despite multiple attempts, we were unsuccessful in detecting eNOS phosphorylation at these residues. Thus, it is unclear whether prenatal methamphetamine causes changes in eNOS phosphorylation in PVAT of the adult aorta.

Recent work by Watts et al. demonstrated that PVAT enhances stretch-induced relaxation of the aorta (20). Aortic rings that are cumulatively stretched over a range of passive tensions exhibit significantly greater relaxation when PVAT is intact than when PVAT is removed (20). Consistent with the work of Watts et al., we found that mechanical stretching of aortic rings (over the range of 0.25–6 g of passive tension) resulted in significantly greater relaxation when PVAT was intact than when PVAT was removed (Supplementary Figure 1). We also found that the ability of PVAT to promote stretch-induced relaxation was unaffected by prenatal methamphetamine. Our finding that prenatal methamphetamine attenuates acetylcholine-induced relaxation without altering stretch-induced relaxation indicates that prenatal methamphetamine selectively alters cholinergic receptor-mediated signaling mechanisms within PVAT while leaving PVAT function associated with mechanical stretching of the tissue intact.

Angiotensin II-induced contractile responses were significantly potentiated in adult male aortas (Figure 3A, Table 3) [but not adult female aortas (Figure 3C, Table 4)] following prenatal exposure to methamphetamine, regardless of whether PVAT was present. However, prenatal exposure to methamphetamine had no impact on angiotensin II-induced contractile responses when nitric oxide synthase was blocked by L-NAME (Figure 3B, Table 3), indicating that potentiation of angiotensin II-induced contraction resulted from methamphetamine-induced changes in NO-dependent signaling. Angiotensin II receptors located on endothelial cells are coupled to the activation of endothelial nitric oxide synthase (eNOS) (21–24). AT1 (21, 22) and AT2 (23, 24) receptor subtypes have both been implicated in NO production in endothelial cells from various arteries, but AT2 receptors are coupled to NO generation in endothelial cells of the thoracic aorta (23, 24). Angiotensin II-induced NO release from the endothelium suppresses the angiotensin II-induced contractile response that is mediated by AT1 receptors located in vascular smooth muscle (22). Taken together, these data suggest that angiotensin II – induced NO release from the endothelium is blunted following chronic fetal exposure to methamphetamine. This loss of NO-dependent relaxation leaves the angiotensin II-induced contractile response in vascular smooth muscle unopposed, resulting in potentiation of angiotensin II-induced contraction (Supplementary Figure 2).

PVAT and the endothelium are both important sources of NO (14, 25). We found that acetylcholine-induced relaxation was unaffected by prenatal methamphetamine when PVAT was removed from the aorta (Figure 2A), suggesting that prenatal methamphetamine had no impact on acetylcholine-induced NO signaling in the endothelium. In contrast, our data suggest that angiotensin II-induced NO signaling in the endothelium was blunted by prenatal methamphetamine (Figure 3). AT2 receptors in the endothelium of the thoracic aorta are coupled to eNOS through a Gαi/adenylate cyclase/protein kinase A-dependent pathway (23, 24). In contrast, endothelial M1 and M3 cholinergic receptors regulate eNOS through Gαq signaling (26, 27). This difference in receptor signaling pathways within the endothelium could provide a mechanism by which prenatal methamphetamine suppresses angiotensin II-induced NO signaling without suppressing acetylcholine-induced NO signaling in the endothelium. However, this is somewhat speculative as we did not measure angiotensin II- or acetylcholine-induced responses in the absence of endothelium.

Previous investigators reported that angiotensin II-induced contraction of the aorta and mesenteric arteries was potentiated in adult male rats following prenatal exposure to nicotine (28). This was attributed to nicotine-induced changes in AT1 (increased expression) and AT2 (decreased expression) receptor expression (measured by western blotting) and was accompanied by an increased thickness of the medial layer of the arterial wall (29). Subsequent work demonstrating that the antibodies used in that study were not specific for the AT1 and AT2 receptors cast doubt on this mechanism (30–32). We considered the possibility that potentiation of angiotensin-induced contraction following chronic fetal exposure to methamphetamine was the result of changes in the expression of AT1 and AT2 receptors. We measured mRNA transcripts encoding AT1a, AT1b, and AT2 receptor subtypes by quantitative polymerase chain reaction and found no methamphetamine-induced changes in the transcripts encoding these receptors (Supplementary Figure 3). We also found no change in the arterial wall thickness of male offspring that had been prenatally exposed to methamphetamine (Supplementary Figure 4). In contrast to prenatal methamphetamine, prenatal nicotine potentiated angiotensin II-induced contraction through a mechanism that was not affected by inhibition of nitric oxide signaling. Thus, prenatal exposure to methamphetamine and prenatal exposure to nicotine potentiate angiotensin II-induced contractile responses in the male aorta through different mechanisms.

Although fetal methamphetamine exposure attenuated acetylcholine-induced relaxation of the adult male aorta, it did not alter acetylcholine-induced relaxation of third order mesenteric resistance arteries which play a role in the regulation of blood pressure. PVAT in the thoracic aorta is primarily brown fat, while PVAT in the mesenteric vasculature is primarily white fat (33, 34). It is possible that prenatal methamphetamine differentially influences the ability of brown and white PVAT to promote acetylcholine-induced relaxation. Watts et al. demonstrated that PVAT has the ability to take up (35), release (36), and metabolize (37) norepinephrine. Methamphetamine inhibits norepinephrine uptake, induces norepinephrine release, and inhibits monoamine oxidase-mediated metabolism of norepinephrine in sympathetic neurons (38, 39). It is unknown whether methamphetamine exerts these same effects on norepinephrine in PVAT. However, prior work has demonstrated that brown fat located in the thoracic aorta contains a much higher (~10-fold) concentration of norepinephrine than the white fat that surrounds mesenteric resistance arteries (40). This could potentially make brown adipose tissue more susceptible than white adipose to the effects of methamphetamine.

The ability of fetal exposure to central nervous system (CNS) stimulants to alter vascular function in adult offspring was the topic of a recent review (41). Chronic fetal exposure to cocaine decreases pressure-dependent myogenic contraction of coronary arteries (10), attenuates acetylcholine-induced relaxation of mesenteric arteries (9), potentiates norepinephrine induced contraction of mesenteric arteries (9), and potentiates the norepinephrine-induced increase in blood pressure (9). Prenatal exposure to nicotine potentiates angiotensin II-induced vasoconstriction in the aorta and mesenteric arteries of adult offspring (28, 42), leads to thickening of the tunica media in the arterial wall (28), attenuates acetylcholine-induced vasodilation (43), and leads to elevated basal blood pressure in adult rats (44) and mice (45). Potentiation of phenylephrine-induced contraction of mesenteric arteries and phenylephrine-induced increase in blood pressure have also been reported in adult rats following chronic fetal exposure to caffeine (46). Furthermore, some of these effects are sex-dependent and have been found exclusively in male animals (9, 10, 47). Our finding that methamphetamine exposure during pregnancy alters vascular function in adult male offspring is consistent with previous work with other CNS stimulants.

Our data indicate that multiple mechanisms (both PVAT-dependent and PVAT-independent) lead to sex-dependent alterations in vascular function following prenatal exposure to methamphetamine. Reactive oxygen species (ROS) such as superoxide can quench nitric oxide without altering eNOS activity or expression (48). Thus, prenatal exposure to methamphetamine might attenuate acetylcholine-induced relaxation through a mechanism that involves ROS-dependent quenching of PVAT-derived nitric oxide. Likewise, quenching of angiotensin II-induced nitric oxide production in the endothelium may be responsible for potentiation of the angiotensin II-induced contractile response in male aortas. In support of this hypothesis, previous work demonstrated that adult rats that were prenatally exposed to nicotine exhibited increased expression of NADPH oxidase 2 and decreased expression of superoxide dismutase in the wall of the aorta (43). These changes resulted in increased oxidative stress in the vasculature. Similar to the findings of the present study, these animals exhibited exaggerated contractile responses to angiotensin II and impaired acetylcholine-induced relaxation. Although methamphetamine is well known for acutely increasing oxidative stress, it is unknown whether prenatal exposure to methamphetamine increases oxidative stress in the adult offspring in the same way that has been reported in adult rats that were exposed to prenatal nicotine. Further work is needed to understand the molecular mechanisms by which prenatal exposure to methamphetamine alters vascular function in the male offspring.

It is unclear why methamphetamine-induced changes in vascular function occurred exclusively in male offspring. Other investigators have reported that fetal hypoxia (49, 50), fetal undernutrition (51), and fetal exposure to maternal diabetes (52) also induce vascular dysfunction selectively in adult male offspring. These male specific effects might reflect greater sensitivity of the developing male vasculature to intrauterine stress in general rather than a sex difference that is specific to intrauterine methamphetamine. Others have reported that fetal cocaine (10), nicotine (53), hypoxia (54), and oxidative stress (55) induce epigenetic changes (mediated by microRNAs and DNA methylation) in gene expression in the vasculature of the adult offspring. However, potential sex differences in these epigenetic effects have not been well studied. Further work is needed to understand the extent to which biological sex influences changes in vascular gene expression that are induced by prenatal exposure to methamphetamine and other forms of intrauterine stress.

In conclusion, this is the first study to demonstrate that chronic fetal exposure to methamphetamine alters the vasculature in adult offspring by inducing PVAT dysfunction and by selectively potentiating angiotensin II-induced vasoconstriction. The concept that prenatal methamphetamine exposure causes long-term changes in vascular function is consistent with our prior work in the heart (8) and the work of other investigators that have identified changes in the adult cardiovascular system following chronic fetal exposure to cocaine (9, 10, 56–59), nicotine (42, 43, 60), and caffeine (46, 61–63). Collectively, these data suggest that chronic fetal exposure to methamphetamine and other stimulants may predispose adult offspring to cardiovascular diseases. Further work is needed to understand the mechanism by which prenatal exposure to these stimulants produce cardiovascular effects in adult offspring in a sex-dependent manner. Work in this field has been limited almost exclusively to rodent studies. Long-term transgenerational studies in humans are needed to determine whether prenatal exposure to methamphetamine and other CNS stimulants alters cardiovascular function in adult human offspring.

## Data Availability Statement

The original contributions presented in the study are included in the article/Supplementary Material, further inquiries can be directed to the corresponding author/s.

## Ethics Statement

The animal study was reviewed and approved by Marshall University Institutional Animal Care and Use Committee.

## Author Contributions

The study was designed by BR. Experiments were performed by HC, AB, DB, and BR. All authors contributed to writing of the manuscript.

## Funding

This work was supported by the National Institutes of Health [R15HL145546].

## Conflict of Interest

The authors declare that the research was conducted in the absence of any commercial or financial relationships that could be construed as a potential conflict of interest.

## Publisher's Note

All claims expressed in this article are solely those of the authors and do not necessarily represent those of their affiliated organizations, or those of the publisher, the editors and the reviewers. Any product that may be evaluated in this article, or claim that may be made by its manufacturer, is not guaranteed or endorsed by the publisher.

## Abbreviations

AT_1A_: angiotensin II type 1A receptor
AT_1B_: angiotensin II type 1B receptor
AT_2_: angiotensin II type 2 receptor
eNOS: endothelial nitric oxide synthase
GAPDH: glyceraldehyde-3-phosphate dehydrogenase
L-NAME: L-nitro arginine methyl ester
NO: nitric oxide
PVAT: perivascular adipose tissue.
