# Supplementary material for: Prenatal Exposure to Methamphetamine Causes Vascular Dysfunction in Adult Male Rat Offspring
Source: Front Cardiovasc Med. 2022 Jan 26;9:830983. doi: 10.3389/fcvm.2022.830983 (PMC8826446; doi:10.3389/fcvm.2022.830983)
Supplement: Supplementary file 1 [file Data_Sheet_1.docx]

**Supplemental Methods**

**Stretch-induced relaxation**. Stretch-induced relaxation was measured in the presence and absence of PVAT as previously described.^1^ Aortic rings were mounted in the tissue bath with the passive tension initally set to 0.25 grams. Cumulative passive tensions of 0.25, 0.5, 1, 2, 4 and 6 grams were applied to the tissue. After each adjustment of tension, the tissue was allowed to relax for 30 min and the tension was recorded. The tissue was then treated with 10 µM phenylephrine to ensure that the tissue was still functional and had not been stretched beyond the physical limits of the contractile machinery. The tissue was then repeatedly washed with Krebs solution over a 30 min period to remove the phenylephrine before adjusting the passive tension to the next level. This process was repeated up to a final passive tension of 6 grams.

**Quantitative polymerase chain reaction:** RNA was extracted from the aortas (with PVAT removed) and the abundance of transcripts encoding angiotensin-II receptors [Angiotensin receptor subtype 1a (AT1aR), angiotensin receptor subtype 1b (AT1bR) and angiotensin receptor subtype 2 (AT2R)] was determined by quantitative polymerase chain reaction. Total RNA was isolated using Trizol (Thermo Fisher, Waltham, MA) according to the manufacturer’s instructions. The RNA was dissolved in 20 µL nuclease free water and the total RNA concentrations were measured using a Nanovue spectrophotometer (GE Healthcare, USA). cDNA was synthesized using 323 ng of total RNA with Superscript^TM^ VILO^TM^ cDNA synthesis kit (Thermofisher, Waltham, CA). The Bio-Rad CFX96 Real-Time PCR Detection system (Bio-Rad Laboratories, Inc., Hercules, CA) was used for quantifying mRNA transcripts encoding angiotensin II receptor subtypes [ThermoFisher catalog # 4331182; Assay ID Rn02758772-s1 (AT1a), Assay ID Rn02132799_s1 (AT1b), Assay ID Rn00560677_s1 (AT2)] by TaqMan^TM^ single gene expression assays (Thermofisher Scientific, Foster City, CA). The following qPCR protocol was used: 95°C for 30 sec (enzyme activation), followed by 40 cycles of 95°C for 15 sec (denaturation) and 60°C for 30sec (annealing/extension), finally a melt curve at 65°C - 95°C. Alien RNA QRT-PCR Inhibitor Alert (catalog no. 300600) obtained from Agilent Technologies (La Jolla, CA) was used as a spiked in reference and for normalization of the curves. PCR was performed in duplicate, and the threshold cycle numbers were averaged.

**Specimen preparation and histology:** Segments from the descending thoracic aorta of 5-month-old male rats were fixed in 10% neutral buffered formalin at room temperature for 48 hours. After fixation, aortas were placed in 70% ethanol for storage. The aorta samples were then processed in an automatic benchtop tissue processor (Leica TP120, Leica Biosystems, Buffalo Grove, IL) and embedded in Surgipath Paraplast Xtra (Leica Biosystems). Embedded aortic segments were cut at 5 µM thickness with a manual rotary microtome (HistoCore BIOCUT, Leica Biosystems). The paraffin-embedded sections were then stained with Gills’ Hematoxylin III (Sigma-Aldrich, St. Louis, MO) and Eosin Y 1% Alcoholic (Astral Diagnostics Inc., West Deptford, NJ) for visualization of general structure.

**Measurement of medial wall thickness:** Stained aortic segments were imaged with a Leica DMi8 inverted microscope (Leica Microsystems, Wetzlar, Germany) and Excelis MPX-20RC color camera (ACCU-SCOPE, Commack, NY) using CaptaVision+ 2.1.2 software (ACCU-SCOPE). Medial thickness was assessed using ImageJ 1.53 software (National Institute of Health, Bethesda, MD). Thickness of the medial wall was determined by averaging the length at 3 points per section of the aorta with a total of 8 sections for each individual rat to give a final average measurement. Average medial thickness measurements were compared between male rats either prenatally treated with methamphetamine or saline using a two-tailed unpaired t test (GraphPad Prism 9.3.0, GraphPad Software, San Diego, CA).


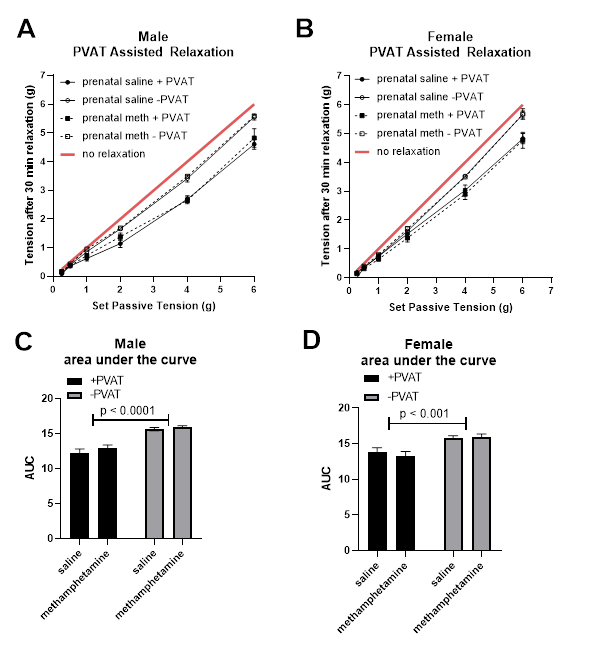


**Supplemental Fig. 1. Prenatal methamphetamine has no impact on PVAT assisted stretch-induced relaxation in the thoracic aorta**. Cumulative tension-relaxation curves were generated in aortas from male (**A**) and female (**B**) rats in the presence or absence of PVAT. The solid bold (red) line indicates the amount of tension in the absence of any relaxation. Two way ANOVA indicated a significant effect of PVAT on the area under the curve in male [F = 65 (1, 12), p < 0.0001] (**C**) and female [F = 21 (1, 12), p < 0.001] (**D**) aortas.


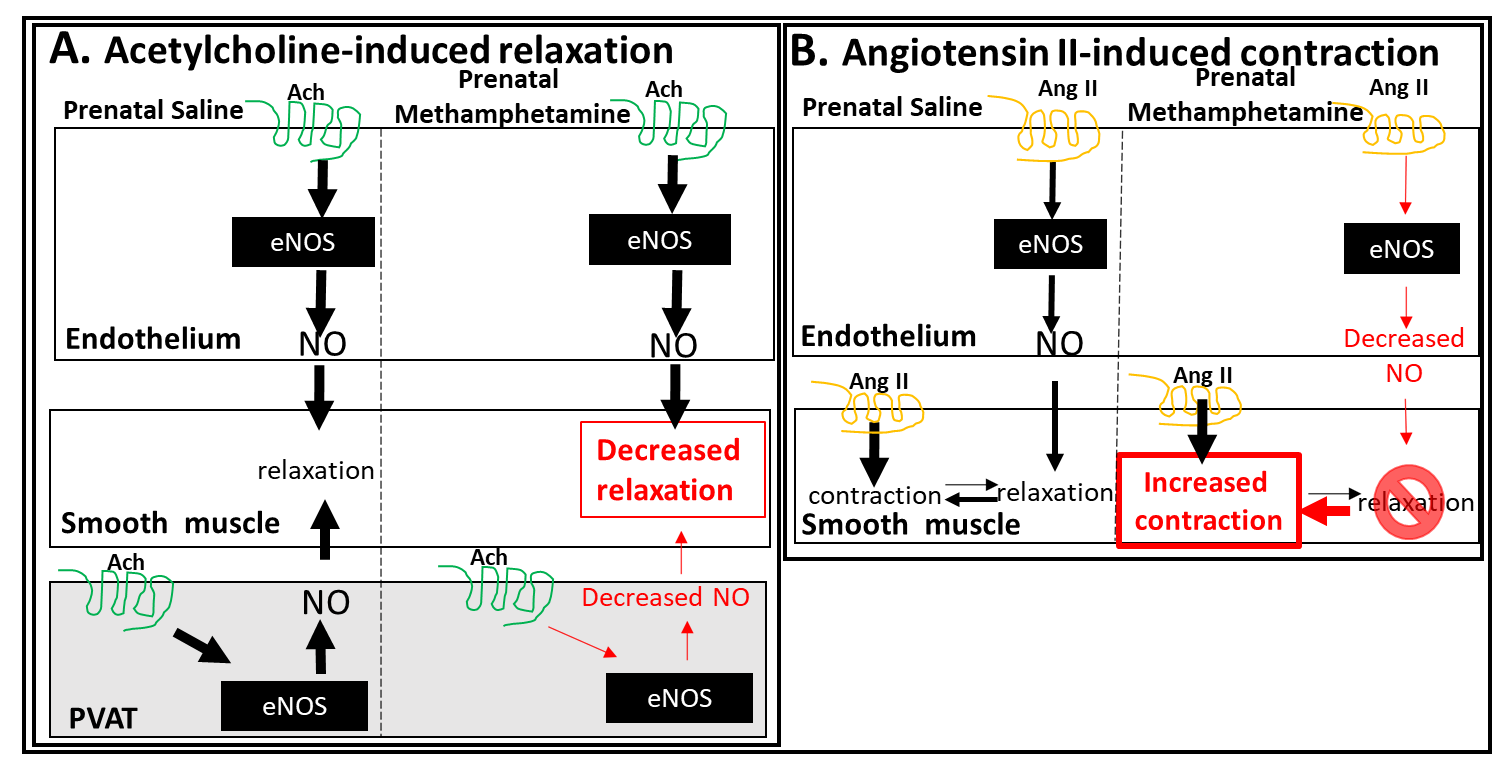


**Supplemental Fig. 2. Potential mechanisms responsible for methamphetamine-induced changes in vascular function in adult male offspring**. Prenatal methamphetamine decreases acetylcholine-induced nitric oxide (NO) production in perivascular adipose tissue (PVAT) of adult male offspring. This results in decreased acetylcholine-induced relaxation of vascular smooth muscle (**A**). In saline treated rats, angiotensin II-induced NO production in the endothelium opposes the contractile response that is mediated by angiotensin II receptors in vascular smooth muscle. Angiotensin II-induced NO signaling is decreased in the endothelium of male rats that were prenatally exposed to methamphetamine. Consequently, less NO is available to oppose to the contractile effect of angiotensin II in vascular smooth muscle. This results in potentiation of the angiotensin II-induced contractile response (**B**). Methamphetamine-induced changes are indicated in red.

**Supplemental Fig. 3. Prenatal methamphetamine has no effect on the number of mRNA transcripts encoding angiotensin II receptor subtypes in the aorta**. mRNA transcripts encoding AT1A (**A**), AT1B (**B**), and AT2 (**C**) receptor subtypes were measured in male aortas by quantitative polymerase chain reaction. Data represent the mean ± S.E.M of aortas from 5 animals.

**Supplemental Fig. 4. Prenatal methamphetamine has no impact on medial wall thickness in the aortas of adult offspring.** Aortas from 5-month-old male rats were photographed following hematoxylin and eosin staining. Photographs are representative examples of aortas from 4 animals in each group taken under 630 X magnification. Scale bars indicate 50 μm.

**References For Supplemental Data**

1. Watts SW, Flood ED, Garver H, Fink GD, Roccabianca S. A New Function for Perivascular Adipose Tissue (PVAT): Assistance of Arterial Stress Relaxation. *Scientific reports*. Feb 4 2020;10(1):1807. doi:10.1038/s41598-020-58368-x
